# Leiomyoma with Uncommon Localization—Incisive Papilla and Palatal Fibromucosa: A Case Report

**DOI:** 10.3390/medicina59071346

**Published:** 2023-07-23

**Authors:** Marina Rakitovan, Adrian Nicoara, Raluca Maria Closca, Nicolae Constantin Balica, Eugen Horatiu Stefanescu, Flavia Baderca

**Affiliations:** 1 Department of Microscopic Morphology, University of Medicine and Pharmacy “Victor Babes”, 300041 Timisoara, Romania; marina.rakitovan@umft.ro (M.R.); raluca.moaca@umft.ro (R.M.C.); baderca.flavia@umft.ro (F.B.); 2 Oro-Maxillo-Facial Surgery Clinic of the Emergency City Hospital, 300062 Timisoara, Romania; nicoara.adrian@umft.ro; 3 Discipline of Dentoalveolar Surgery, University of Medicine and Pharmacy “Victor Babes”, 300041 Timisoara, Romania; 4 Department of Otorhinolaryngology, University of Medicine and Pharmacy “Victor Babes”, 300041 Timisoara, Romania; stefanescu@umft.ro; 5 Otorhinolaryngology Clinic, Emergency City Hospital, 300054 Timisoara, Romania; 6 OftalmoSensory-Tumor Research Center-ORL (EYE-ENT), University of Medicine and Pharmacy “Victor Babes”, 300041 Timisoara, Romania; 7 Service of Pathology, Emergency City Hospital, 300254 Timisoara, Romania

**Keywords:** oral pathology, leiomyoma, incisive papilla, palatal fibromucosa, surgical excision, immunohistochemical reaction

## Abstract

The current paper presents a case of a 33-year-old female with an uncommon localization of a leiomyoma in the oral cavity—the anterior palatal fibromucosa and the incisive papilla. The patient referred to the Oro-Maxillo-Facial Surgery Clinic of Emergency City Hospital Timisoara, Romania, complaining of a slight discomfort in the act of mastication and the occurrence and persistence of a diastema between the upper central incisors, due to the presence of a nodule located in the anterior palatal mucosa, between the upper central incisors, without any changes of the subjacent bone structure in the anterior hard palate visible on a cone beam computed tomography image (CBCT). The lesion was removed using a surgical excisional biopsy and a histopathological examination was performed using morphological Hematoxylin–Eosin (HE) staining and additional immunohistochemical (IHC) reactions, in order to confirm the diagnosis. On microscopic examination, bundles of spindle cells were found with eosinophilic cytoplasm and vesicular nuclei, with finely granular chromatin. The immunohistochemical reactions were positive for smooth muscle actin (SMA) and desmin and negative for vimentin. The treatment of choice for leiomyoma of the oral cavity is surgical excision with clear margins, followed by periodical clinical monitoring.

## 1. Introduction

Leiomyoma is a benign tumor of the smooth muscle, mostly found in the uterus, gastrointestinal tract, skin and subcutaneous tissues, with a rare incidence in the pathology of the oral cavity [1,2,3,4]. Oral leiomyomas present themselves as progressive, slow-growing, asymptomatic masses [1,2,3,5,6], mostly found in the lingual interstitium, labial mucosa, fibromucosa of the hard palate or buccal mucosa [1,5,7]. The smooth muscle cells retain the mitotic capacity during the lifetime of humans; therefore, the occurrence of benign muscular tumors of the muscular tunica in different organs such as uterus and gastrointestinal tract segments is not unexpected. At the skin level, the origin of cutaneous leiomyomas is represented by hair erector muscle or the tunica media of the blood vessels. The scarcity of smooth muscle tissue in the structures of the oral cavity region can be an explanation for the low occurrence of leiomyomas in this anatomical region [1,6].

They may appear at any age, in both sexes, but are usually discovered when the dimensions get visible and palpable. A certain diagnosis is determined using histopathological examination performed under special staining that can confirm the smooth muscle origin [5]. The treatment for this type of tumor is surgical removal [1,2,3,4,5,6,7,8,9]. If radical excision with free margins is obtained, the recurrence of the tumor is mainly unexpected [1,2,3,4,5,7,8,9,10]; nonetheless, periodical clinical monitoring is highly recommended.

In this article, we present a case of a 33-year-old female with an uncommon localization of a leiomyoma—the incisive papilla and anterior palatal fibromucosa of the oral cavity—in order to bring awareness towards a rare etiology of a diastema.

## 2. Materials and Methods

### Extensive Case Presentation

The current paper is a retrospective presentation of a 33-year-old female patient, without significant conditions nor diseases throughout her life (bronchitis in childhood, *Helicobacter pylori* infection treated with medication), referred to the Oro-Maxillo-Facial Surgery Clinic of Emergency City Hospital Timisoara, Romania, in March 2021, complaining of a slight discomfort in the act of mastication and the occurrence and persistence of a diastema between the upper central incisors, due to the presence of a small tumefaction located in the anterior hard palate, with the involvement of the incisive papilla and extended between the upper central incisors. Anamnestic, the patient established the occurrence of the lesion approximately 8 years prior, with a continuous, slow, progressive growth.

**Figure 1:**
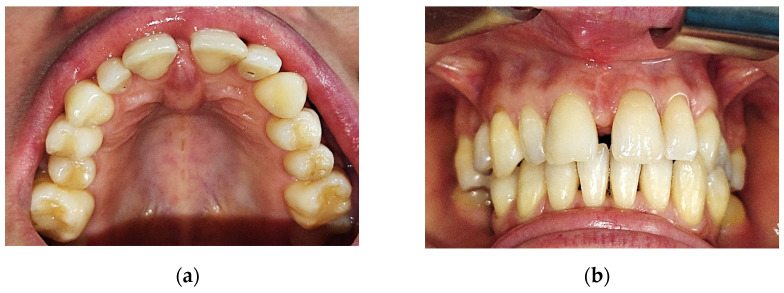
Intraoral aspect: (**a**) aspect of the lesion at the level of the incisive papilla and anterior palatal fibromucosa; (**b**) aspect of the lesion (buccal view), interincisal diastema and gingivo-mucosal scar.

At the clinical exam, the lesion had a shape of a droplet, well delimited, with a dimension of approximately 15 mm (sagittal plane)/10 mm (transverse plane), with a nodular lower pole located at the median palatal fibromucosa, extended between the bilateral palatal rugae. It had a pink tint, smooth surface and soft, resilient and depressible consistency. The upper pole of the lesion was located interdentally between the superior central incisors (1.1–2.1, using the FDI Dental Numbering System, or 8–9, using the Universal Dental Numbering System). On the buccal side of the alveolar crest, the lesion was in contact with the crestal insertion of a hypertrophic upper labial frenulum, having a reddish color and a slightly firmer consistency, apparently fixed to the underlying bone (Figure 1).

The clinical examination also revealed a dentoalveolar incongruence with the presence of a maxillary interincisal diastema of 2 mm and a slight distal tipping of the right upper central incisor, with delicate coverage, that might be associated with the presence of the mentioned lesion. Furthermore, a median buccal gingivo-mucosal scar presented between the two upper central incisors, 1.1/8–2.1/9. The patient affirmed a previous surgical intervention for a frenoplasty of the upper labial frenulum, 3 years before the current hospital presentation, conducted in a private dental office, with intension to correct the diastema, which had no benefits.

**Figure 2:**
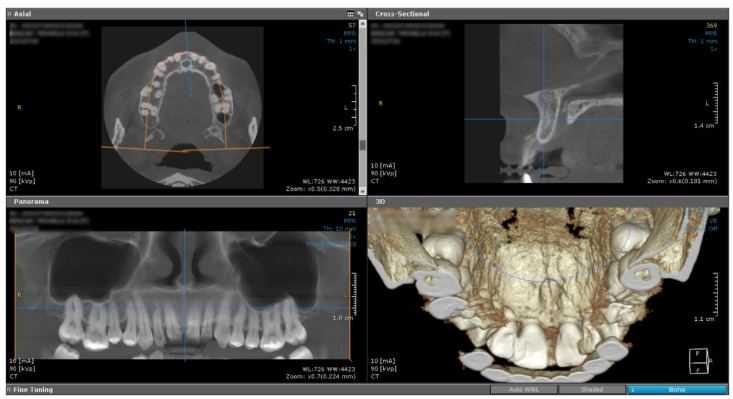
Cone beam computed tomography showed no underlying bone structure alteration.

A cone beam computed tomography image (CBCT) did not reveal any changes of the subjacent bone structure in the anterior hard palate, suggesting the sole involvement of the soft tissue (Figure 2).

Based on clinical and imagistic aspects, a presumptive diagnosis of oral irritation fibroma was established. The surgical removal of the lesion was decided upon. The surgical intervention and the harvest of the lesion were approved by the patient in a written consent.

Under local anesthesia, a classical incision was performed that circumscribed the lesion at the level of the palatal mucosa, as well as the interincisive alveolar ridge. The lesion was easily detached from the underlying bone and removed. The lesion presented no invasiveness in the surrounding tissues. No remaining parts were identified. Hemostasis was achieved using monopolar electrocauterization. The postoperative defect was closed using a marginal–marginal suture at the posterior palatal level by placing non-absorbable suture threads 4.0 separately and a vertical mattress. The residual gingival defect was protected by a periodontal dressing, for the purpose of “per secundam” healing.

The harvested tissue, fixed in 10% (*v/w*) neutral buffered formalin, was sent to the Department of Pathology of Timisoara Emergency City Hospital, for a histopathological examination.

The gross examination of the harvested specimen revealed a 10/10/3 mm white-brownish elastic tissue fragment that was processed without sectioning for histopathological examination. 

Four-micrometer-thick serial sections were prepared for the diagnosis from paraffin blocks, using morphological Hematoxylin–Eosin (HE) staining. 

**Figure 3:**
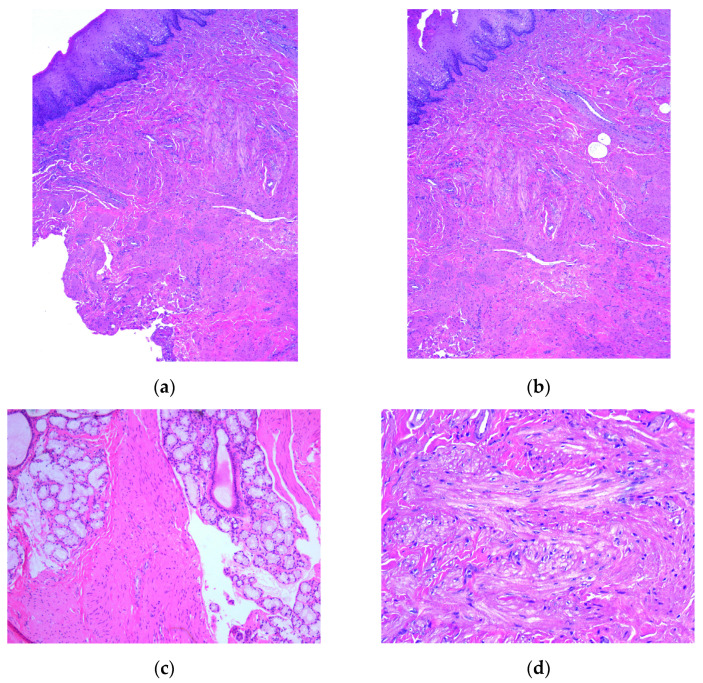
Microscopic aspects of HE-stained slides: (**a**) tumor proliferation in the oral mucosa with spindle-shaped cells arranged in different-sized fascicles, ob. 5x; (**b**) different-sized spindle cell fascicles disposed in the lamina propria of the oral mucosa, ob. 5x; (**c**) fascicles of tumor cells arranged among acinar structures in the lamina propria of the mucosa, ob. 5x; (**d**) fusiform tumor cells in a fasciculate pattern, with large, eosinophilic cytoplasm and elongated monotonous blunt-ended nuclei, ob. 20x.

The microscopic examination of Hematoxylin–Eosin-stained slides revealed that a multilobulated benign tumor developed in the lamina propria of oral mucosa composed of observed different-sized fascicles of spindle-shaped cells, with large eosinophilic cytoplasm and vesicular, monotonous, blunt-ended nuclei, with finely granular chromatin. The spindled cells infiltrated the normal structure of buccal mucosa as connective tissue, adipose lobules, seromucous acini and glandular ducts (Figure 3). Based on the histopathological aspects of HE-stained slides, a presumptive diagnosis of a spindle-shaped-cell benign tumor (fibroma or leiomyoma) was raised. 

Therefore, immunohistochemical (IHC) reactions for vimentin, smooth muscle actin (SMA) and desmin were performed. Moreover, the mitotic index was established using Ki67 antibodies. 

All the data regarding the antibodies used for IHC reactions are centralized in Table 1. The panel of antibodies (Anti-Desmin Antibody, Anti-SMA Antibody, Anti-Vimentin Antibody and Anti-Ki67 Antibody) and the reagents utilized for immunohistochemistry were acquired from Novocastra™ and Bond™, respectively, Leica Biosystems.

**Figure 4:**
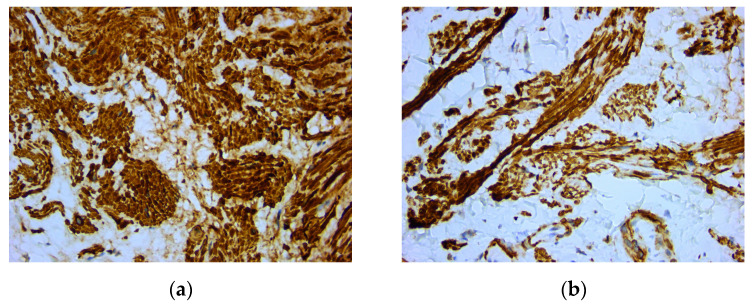
Microscopic images of IHC reactions for desmin, ob. 40x: (**a**) tumor cells with diffuse and intense positive reaction for desmin; (**b**) intense and diffuse cytoplasmic positive reaction in all the tumor cells.

**Figure 5:**
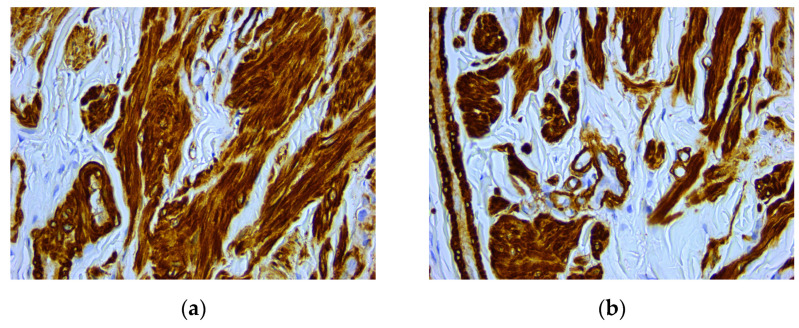
Microscopic images of IHC reactions for smooth muscle actin, ob. 40x: (**a**) intense and diffuse positive reaction in all tumor cells; (**b**) positive reaction with cytoplasmic distribution.

**Figure 6:**
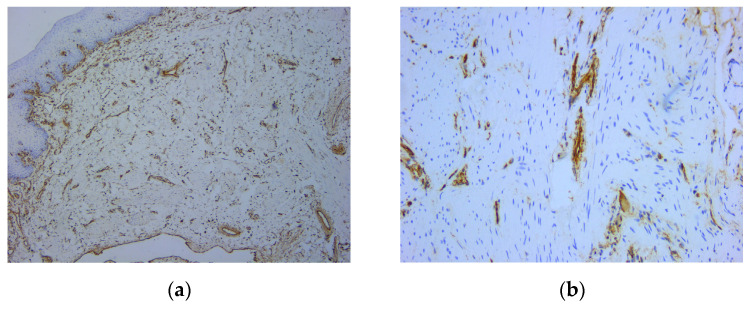
Microscopic images of IHC reactions for vimentin: (**a**) negative reaction of tumor cells, with positive control, ob. 5x; (**b**) positive reaction at the level of the vascular component, ob. 20x.

**Figure 7:**
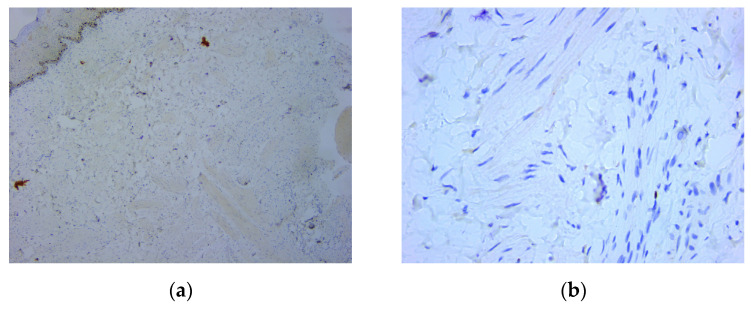
Microscopic images of IHC reactions for Ki67: (**a**) positive reaction in the nuclei of basal layer cells of the covering epithelium, ob. 5x; (**b**) only a few positive nuclei of the tumor cells, ob. 40x.

An intense and diffuse positive immunohistochemical reaction in all the tumor cells was found for desmin with a cytoplasmic pattern (Figure 4). In addition, smooth muscle actin was intensely positive in all the tumor cells with a cytoplasmic and nuclear pattern of distribution (Figure 5). On the other hand, vimentin was negative, with positive internal control at the level of the connective tissue (Figure 6). The Ki67 cell proliferation index was 3%, with only a few tumor cells being positive (Figure 7). 

Given the histopathological aspects on HE-stained slides and correlation with immunohistochemical reactions, the final diagnosis of leiomyoma was signed off on. 

The patient was informed of the final diagnosis and that the therapeutic plan for this type of tumor is exclusively surgical. Therefore, no further treatment was necessary; the patient was asked to come for periodical check-ups, at which no significant postsurgical events were noted.

## 3. Discussion

Leiomyoma is a benign smooth muscle tumor that can develop in any site [1,3,9]. The most frequent area is represented by the female genital tract (95%), followed by the skin (3%) and gastrointestinal tract (1.5%). Even if it is a relatively common tumor, in the oral cavity, leiomyoma is not encountered that often [2,3,5,6,7,10]. Less than 1% out of the total number of leiomyomas arise in the head and neck region, while only 0.065% occur in the oral cavity [1,4,9,11]. Meanwhile, leiomyoma represents less than 1% of all tumors of the oral cavity [2].

Benign tumors of the smooth muscle are rarely present in the oral cavity and are usually non-aggressive [2,6].

The earliest report of an oral leiomyoma was by Blanc in 1884 [10]. The sporadic presence in the oral cavity, indistinguishable clinical appearance and, nonetheless, variable histopathological images can often lead to a misdiagnosis, with numerous differential diagnoses [1,2,10]. Da Silva et al. demonstrated that this benign tumor accounted for only 0.9% of 790 oral soft tissue neoplasms [12].

Due to the scarcity of the smooth muscle in the structures of the oral cavity [1,10], the origin of leiomyoma in this area is limited to the following possible sites: tunica media of the blood vessels, ductus lingualis, circumvallate papilla [3,10] or heterotopic embryonic tissue [5,10].

Oral cavity leiomyoma may appear at any age, but most authors reported that this pathology is usually present in adults, with the greatest incidence in the fourth and fifth decade of life [1,3,5,6,10]. The present article reports a case of a 33-year-old female patient in the moment of the diagnosis, affirming the presence of the lesion for 8 years. The appearance of the tumor was in the patient’s early twenties, which presents quite a distance from the published literature.

Regarding the gender distribution of oral leiomyoma, there is not one common opinion shared by authors. Some of them claimed that there is a male predominance of oral leiomyoma [3,5,10,11,13] and others argued that it is more frequent in female patients [1,6], while other authors stated a similar distribution in both sexes [2,9,10].

The most common sites of the occurrence of leiomyoma in the oral cavity are the lingual, labial, hard or soft palate and jugal mucosa [2,8]; cases were also outlined in other less frequent locations, such as the retro molar trigon, floor of the mouth, gingiva or submandibular region [3,5]. Additionally, cases with the intraosseous localization of leiomyoma with the involvement of the jaws, mainly in the mandible bone, were also reported in the literature [2,6,14].

In the English literature, there is no other article that presents a tumor with an anterior hard palate localization, as shown in our case. 

There is a general agreement between authors regarding the following most common clinical characteristics of leiomyoma affecting oral cavity structures, especially the ones involving the hard palate: small (<3 cm), solitary, slow-growing nodular mass and firm to the touch [1,2,3,5,6,9,10,12]. For the majority of the reported cases of oral cavity leiomyoma, the color of the lesion had a pinkish, reddish, bluish, grayish or purplish tint [2,8,9,13], depending on their depth and vascularity [5], with a surface that resembles the texture of the normal neighboring mucosa [6,10]. 

In our case, the characteristic of the tumor were overlapping with those of the literature.

Generally, leiomyoma of the oral cavity is characterized as an asymptomatic lesion [1,3,5,6,7] but in some cases, as the tumor evolves, some symptoms may occur, such as pain, teeth mobility, toothache or even difficulty in chewing or deglutition [1,3,5,6,8,10].

In our case, the patient did not present any symptoms other than a slight discomfort in the act of mastication and the occurrence and persistence of a diastema between the upper central incisors, where the anterior apex of the tumoral lesion in discussion here was inserted interdentally. For this dentoalveolar incongruence, the patient sought prior dental treatment and a frenoplasty procedure was performed. The persistence of the diastema was the main reason for the patient being referred for surgical treatment, regarding an “enlarged incisive papilla inserted on the alveolar crest”. The misdiagnosis, followed by repeated medical procedures that attempted to treat the complication, not the cause, were the reason for the 8-year-delay between the occurrence of the lesion and the time of surgical excision, with a definitive histopathological diagnosis of the tumor. This outcome, in our opinion, might be explained up to a point by the lack of pain or other important symptoms, the slow-growing characteristic of the leiomyoma and the quasinormal aspect of the tumoral surface, characteristics that can lead to difficulties in a clinical diagnosis, postponed surgical treatment and a final histopathological diagnosis. 

While the clinical appearance of oral leiomyoma is unspecific [1,2,3,5], the final diagnosis is established using histopathological examination, with specific immunohistochemical staining for a smooth muscle origin [1,2,3,5].

From the clinical point of view, the differential diagnosis of oral leiomyoma is very difficult and should include other benign tumors (fibroma, neurofibroma, lipoma, etc.), salivary gland tumors (mucocele, pleomorphic adenoma), vascular tumors (lymphangioma, hemangioma) and even tumors of the periodontium, but what is of the upmost importance is to differentiate leiomyoma from the malignant counterpart, leiomyosarcoma [1,4,5,10].

A histopathological diagnosis can sometimes involve difficulties in differentiating leiomyoma from other tumors; for example, schwannoma (neurilemmoma), neurofibroma, myofibroma, myopericytoma/hemangiopericytoma, solitary fibrous tumors, benign fibrous histiocytoma, spindle cell pleomorphic adenoma or well-differentiated/low-grade leiomyosarcoma [2,4,10]. Leiomyosarcoma is composed of interlacing spindle-shaped-cell fascicles with blunt-ended nuclei and mild or severe atypia [2]. Well-differentiated leiomyosarcoma is a very similar lesion to leiomyoma, except the nuclei are more hyperchromatic and the mitotic activity is prominent. The high Ki67 proliferation index (>10% nuclear staining) is necessary for separating benign from malignant tumors. The final diagnosis can be established only by using immunohistochemical staining that identifies smooth muscle components [10].

There is a general consensus concerning the treatment of oral cavity leiomyoma—that the surgical excision with clear margins and periodical monitorization for observing eventual recurrences is the most successful therapeutical attitude [1,2,3,4,5,6,7,8,9]. In case of the complete resection of the tumor, the recurrences rates are very low, followed by a favorable postoperative prognosis [1,2,3,4,5,7,8,9].

For our patient, the complete resection of the tumor was performed, using a classical incision with a cold scalpel, with a good postoperative outcome, without any relapse at the 3-, 6- and 12-month follow up.

To the best of our knowledge, this is the first article that presents a leiomyoma that affected the incisive papilla and anterior palatal fibromucosa of the oral cavity.

## 4. Conclusions

The paper presented a case of leiomyoma of the oral cavity with an uncommon localization, the midline fibromucosa of the anterior hard palate, involving the incisive papilla, extended on the alveolar ridge between the upper central incisors. Because of its unspecific clinical appearance and, nonetheless, rarity in the oral cavity, establishing a diagnosis can be a laborious task. The final diagnosis depends upon histopathological evaluation with immunohistochemical staining for a smooth muscle origin. The treatment of choice is surgical excision with clear margins, followed by periodical clinical monitoring. All things considered, the reporting of oral leiomyoma cases is meaningful for growing the literature, overcoming histopathological challenges and improving surgical experiences.

## Figures and Tables

**Table 1:** Data related to the antibodies used for immunohistochemical reactions.

Antibody	Substrate	Dilution	Clone
Smooth Muscle Actin (SMA)	Monoclonal Mouse	Ready-To-Use	asm-1
Desmin	Monoclonal Mouse	1:200 for 30 min at 25 °C	DE-R-11
Vimentin	Monoclonal Mouse	1:800 for 30 min at 25 °C	V9
Ki67	Monoclonal Mouse	Ready-To-Use	MM1
